# MIB2 promotes the progression of non-small cell lung cancer by regulating cell cycle control pathways

**DOI:** 10.1007/s13258-023-01423-4

**Published:** 2023-07-12

**Authors:** Yiru Kong, Jing Li, Xiaohua Liang, Xinli Zhou

**Affiliations:** 1 ¹Department of Oncology, Huashan Hospital Fudan University, 12 Middle Urumqi Road, Shanghai, 200000 China; 2 Shanghai Medical College, Fudan University, Shanghai, 200032 China; 3 Department of Oncology, Huashan Hospital Fudan University, 12 Middle Urumqi Road, Shanghai, 200040 China

**Keywords:** MIB2, NSCLC, Cell cycle, Proliferation

## Abstract

**Background:**

Although numerous measures have been used to improve the outcome of lung cancer patients, lung cancer, as the second most common diagnosed cancer, is still the main cause of cancer death. It becomes increasingly urgent for us to deeply deplore the molecular mechanism of lung cancer and to discover the potential therapeutic targets. In our study, we are dedicated to discovering the role of MIB2 in lung cancer development.

**Methods:**

The public databases were used to compare the expression level of MIB2 in cancer and non-cancer tissue. We analyzed the expression of MIB2 in lung cancer samples by performing Rt-PCR and western blot. We carried out CCK8 and clone assays to study the influence of MIB2 in lung cancer proliferation. The transwell assays and wound healing assays were implemented to study the function of MIB2 in metastasis and invasion. Proteins of cell cycle control pathways are detected to verify the potential mechanism of MIB2 in lung cancer progression.

**Results:**

MIB2 is up regulated in lung cancer tissue compared to adjacent normal lung tissue according to both public databases and our clinical lung cancer samples. Knockdown of MIB2 inhibits proliferation, metastasis, and invasion of lung cancer cell lines. Cyclins and cyclin dependent kinases (CDK) including CDK2, CDK4, and cyclinB1 were down regulated in MIB2 knockdown cells.

**Conclusion:**

Our results prove that MIB2 acts as a driver in NSCLC tumorigenesis by regulating cell cycle control pathways.

**Supplementary Information:**

The online version contains supplementary material available at 10.1007/s13258-023-01423-4.

## Introduction

According to the global cancer statistics in 2020, lung cancer, as the second most common diagnosed cancer after breast cancer, is expected to account for 11.4% of the total number of malignant tumors. Meanwhile, lung cancer is still the main cause of cancer death(Sung et al. 2021). Although there has been an increase in the novel therapeutic approaches for lung cancer over the past decades such as several tyrosine kinase inhibitors in patients with EGFR, ALK, ROS1, and NTRK mutations, the 5-year survival rate of patients is still only 21%. The high degree of malignancy, the complex pathogenesis, and the high heterogeneity of lung cancer resulted in endless drug resistance after targeted therapy(Herbst et al. 2018). Clarifying the molecular mechanism of lung cancer and exploring new therapeutic targets are the key scientific issues that need to be solved nowadays.

By analyzing the GEO datasets (GSE 75,037 & 30,219), we finally focused on MIB2 (Mind Boom 2), an E3 ubiquitin protein, was highly expressed in lung cancer samples compared to non-cancer tissue. Consistent results were also obtained in the TCGA database. Besides, MIB2 is also negatively correlated with lung cancer prognosis. Previously, it is reported that MIB2 coordinates with circGlis3, Gm364, and some other molecules in causing cognitive impairment, placenta-related diseases, type 2 diabetes, as well as regulating female fertility(Chen et al. 2022; Lin et al. 2021; Xiong et al. 2022; Zhao et al. 2021). Deletion of MIB2 is reported to have something to do with Cowden syndrome, Ménétrier-like gastropathy, and may help to alleviate the injury caused by ischemia (Cavaillé et al. 2018; Li et al. 2020; Piccolo et al. 2017). MIB2 may even involve in regulating muscle stability(Carrasco-Rando and Ruiz-Gómez 2008; Nguyen et al. 2007).

Nevertheless, few studies have been performed on the biological effects and molecular functions of MIB2 in lung cancer development. In our research, we demonstrate that MIB2 is highly expressed in lung cancer cell lines and is associated with metastasis, staging, and survival time of non-small cell lung cancer. What is remarkable is that MIB2 knockdown inhibits proliferation, metastasis, and invasion of NSCLC cell lines. More importantly, we provide the evidence that MIB2 is involved in modulating the cell cycle which may be the mechanism related to lung cancer proliferation. Collectively, our study revealed that MIB2 promoted the occurrence of lung cancer by regulating cell cycle, which may help to promote the development of new diagnostic or therapeutic biomarker to improve the prognosis of patients with NSCLC.

## Methods and materials

### Cell culture

Eight human non-small cell lung cancer cell lines including A549, H1299, PC-9, H1975, H358, H292, and H460 were auquired from the American Tissue Culture Collection (ATCC). All cell lines were cultured in accordance with ATCC protocols and were all supplemented with 10% fetal bovine serum (FBS) and 10% penicillin sodium and streptomycin sulfate. All cell lines are cultured at 37 °C, 5% carbon dioxide in the humidified incubator.

### Human NSCLC samples

The 8 pairs of human NSCLC samples (both cancer and the corresponding adjacent normal lung tissue) were obtained from patients going through surgery in the Huashan Hospital Fudan University from 2017 to 2018. The 65 pairs of NSCLC samples were collected from Shanghai Chest Hospital during 2011–2012. All the samples were frozen immediately in liquid nitroge during surgery and stored at the − 80 °C refrigerator. This study was authorized by the Ethics Committee of Huashan Hospital Fudan University. The institutional approval number is 2021 − 922. Written informed consent has been collected from each patient. All procedures were carried out in accordance with the ethical code of the Declaration of Helsinki.

### RNA extraction and real-time polymerase chain reaction (qRT-PCR) assays

TRIzol reagent (Invitrogen, California, USA) was used to isolate the RNA samples from the clinical lung tissue and cell lines used in this research. The PrimeScript RT Reagent Kit (TaKaRa, Dalian, China) was used to extract cDNA synthesis. Real-time polymerase chain reaction (qPCR) was performed through SYBR Green Premix Ex Taq (TaKaRa, Dalian, China) and performed by 7900 Real-time PCR System (Applied Biosystems). β-actin was used as the internal control. The following primer sequences were used:

MIB2: Forward: ACCTGCTGCTGTACGACAAC;

Reverse: GTGCATGTAGCACTGCGTG.

β-actin: Forward: GTCATTCCAAATATGAGATGCGT;

Reverse: GCATTACATAATTACACGAAAGCA.

### Western blot

Tissue Protein Extraction Reagent was used to isolate all protein samples of lung cancer tissue and cell lines that used in this research. Bicinchoninic acid (BCA) Protein Assay Kit was used to measure the protein concentration. Protein lysates extracted from cells were separated by SDS-PAGE gel electrophoresis and transferred to Polyvinylidene Fluoride (PVDF) membrane. Using 5% skimmed milk to block the membrane and probe it with the primary antibodies. Incubating the primary antibodies overnight at 4 °C refrigerator and probing the membrane with species-specific secondary antibodies the next day. The antibodies used are listed as follows: MIB2 (Biodragon, 1:1000; Thermofisher, A301-414 A-T); CDK2, CDK4, CDK6, Cyclin B1(CST, 1:1000), β-actin (Sigma-Aldrich, 1:10000).

### Cell transfection

The small interfering RNA (siRNA) oligonucleotides targeting MIB2 were designed and synthesized by RiboBio (Guangzhou, China). Cells were seeded in the 6-well plates a day before the transfection in 60–70% confluence. Cells were transfected with siRNAs by Lipofectamine 2000 reagents (Invitrogen, USA) in medium with no FBS. The cells used for proliferation, migration, invasion assays were validation by qPCR and western blot after 48 h transfection. Sequences of the siRNAs are shown below:

Si-MIB2#1: GCTACATGCACAACAAGCA.

Si-MIB2#2: GGAGGTGCCAAACATCGAT.

### Lentivirus constructs and transfection

Lentivirus constructs Hemagglutinin-MIB2 were designed and synthesized by RiboBio (Guangzhou, China). To produce the intended lentivirus, the constructed plasmids packaging system was transfected into 293T cells using Lipofectamine 2000. After 48 h, the medium from the 293T cultures were collected to infect PC9 cells.

### Cell proliferation, migration and invasion assays

After seeding in the 96-well plates, the proliferation of A549, H1299, and PC9 were measured by Cell Counting Kit-8 (CCK-8) (Dojindo, Kumamoto, Japan). Cell migration and invasion assays were conducted according to the protocols of Transwell filter chambers (BD Biosciences, New Jersey, USA). For migration assays, all of A549, H1299, and PC9 cells were incubated into the upper chamber per well with serum-free culture. For invasion assays, A549, H1299, and PC9 cells were suspended into the upper chamber with a Matrigel-coated membrane diluted with serum-free culture medium per well. Placing 800-µL culture medium with 10% FBS in the lower chamber. After incubation in incubator at 37 °C overnight, the cells in the bottom surface of the membrane were fixed with 100% methanol and counted under a microscope. For colony formation assay, cells were placed in 6-well plates and cultured for 14 days. Washing the plates with phosphate-buffered saline (PBS) for at least three times. Fixing and staining the colonies for 30 min respectively.

### Cell cycle analysis

Cells used for cell cycle and apoptotic assays were transfected with siRNAs or lentivirus after 48 h. After washed with PBS for three times, cells were fixed with 70% ethanol and placed at -20 °C refrigerator. Staining the cells with 500 µl propidium iodide (PI) with RNAseI next day.

### Wound-healing assays

Both the lung cancer cell lines were seeded in six-well plates by 60% confluence. After 36–48 h of cell transfection, a single scratch wound was created using a 200-µL pipette tip. Then remove the cell debris by washing with PBS at least 3 times. Washing the cell debris as much as possible so as not to interfere with the statistics. Then replace the 10% FBS with 1% FBS culture medium and continue culturing the cell lines. The images were immediately photographed at 0 and 24 h after wounding. The wound size was measured by the software Image J.

### Statistical analysis

Data were presented as mean ± SEM (Standard error of mean), and every representative experiment was repeated three times. Differences between two groups were compared by the unpaired two-tailed Student’s t-test. Graphpad prism version 8.0 software was used to visualize the data. A p-value less than 0.05 was considered statistically significant.

## Results

### MIB2 is upregulated in NSCLC patients and is correlated with advanced clinical stages of patients

**Fig. 1:**
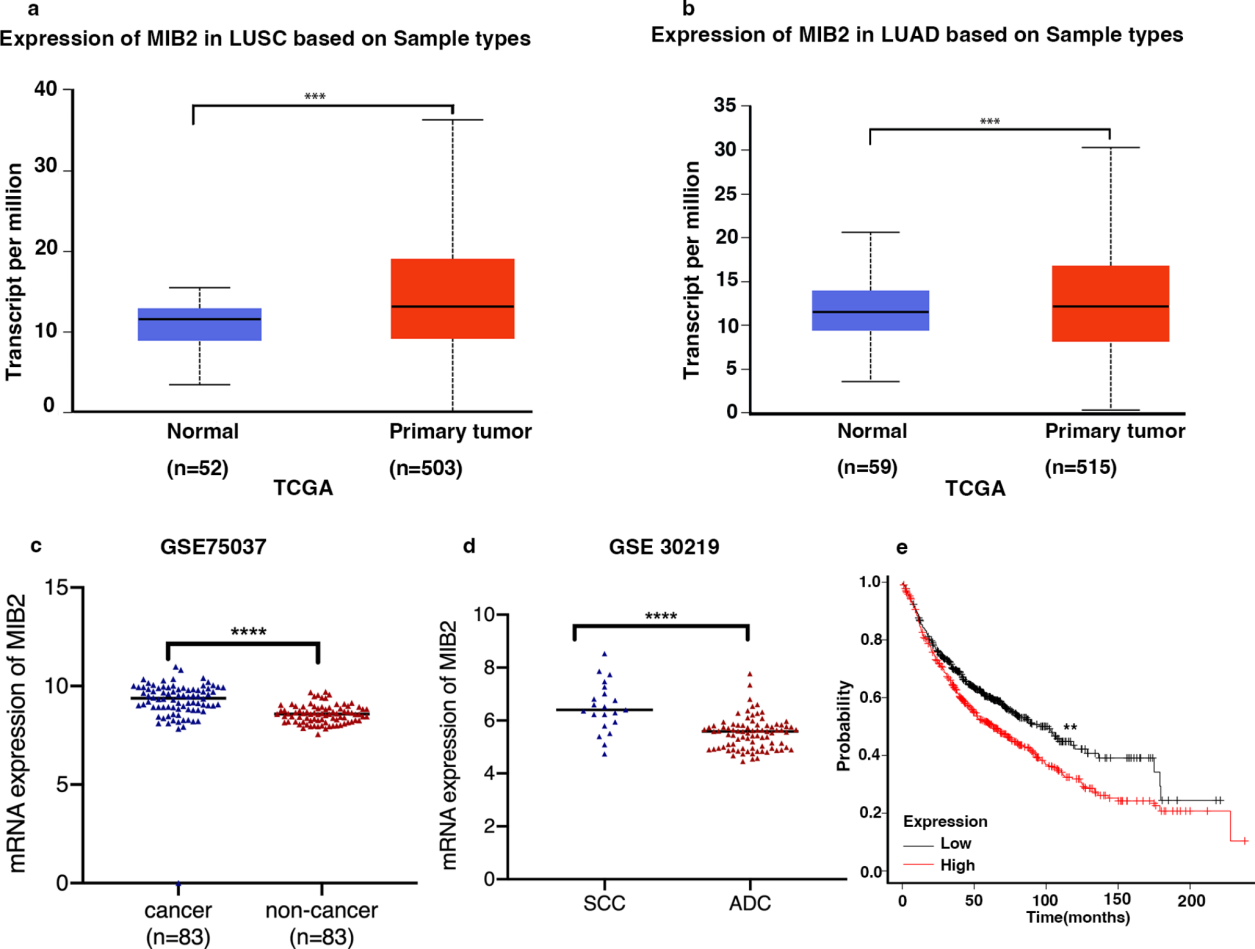
MIB2 expression in public database. (**a-b**) Expression of MIB2 in LUSC and LUAD from TCGA database. (**c**) Real-time PCR analysis from GSE 75,037 to quantify the levels of MIB2 in 83 pairs of NSCLC tissue. (**d**) Real-time PCR analysis from GSE 30,219 to quantify the levels of MIB2 in patients with clinical early-stage (I-II) and advanced-stage (III-IV) NSCLC. (**e**) Kaplan–Meier plot analyses of the correlation between MIB2 expression and overall survival of NSCLC patients from the GEO cohort. Statistical analysis was performed using Student’s t-test; Error bars represent the SEM. *p < 0.05; **p < 0.01; ***p < 0.001

**Fig. 2:**
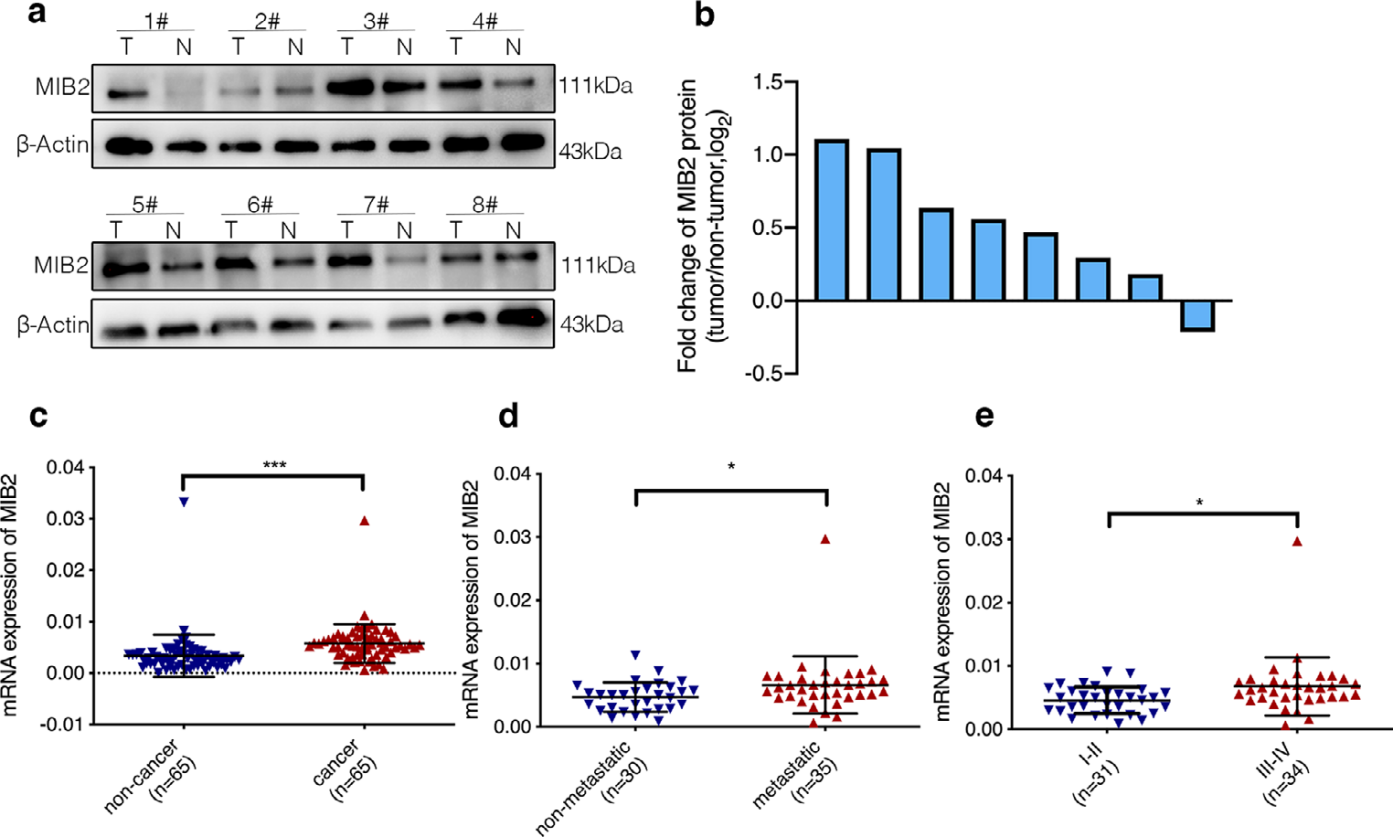
MIB2 expression in clinical samples. (**a-b**) Western blot analysis to quantify the levels of MIB2 in 8 paired NSCLC tissue. (**c**) The levels of mRNA expression of MIB2 in 65 paired NSCLC tissue. (**d**) Real-time PCR analysis to quantify the levels of MIB2 in patients with or without metastasis. (**e**) Real-time PCR analysis to quantify the levels of MIB2 in patients with clinical early-stage (I-II) and advanced-stage (III-IV) NSCLC. Statistical analysis was performed using Student’s t-test; Error bars represent the SEM. *p < 0.05; **p < 0.01; ***p < 0.001

Through mining the public TCGA dataset, we detected that MIB2 is significantly up regulated in both lung adenocarcinoma and lung squamous carcinoma tumor tissue (Fig. 1a and b). Using the public GEO dataset (GSE 30,219 and GSE75037), we observed that MIB2 expression was up regulated in lung cancer tissue and was associated with advanced stages and worse prognosis of lung cancer patients (Fig. 1c and d). Kaplan-Meier plot survival analysis based on GEO database was performed to examine the expression of MIB2 and revealed that the higher expression of MIB2 was correlated with poor outcomes in NSCLC patients (Fig. 1e). To further examine the role of MIB2 in lung cancer, we detected MIB2 expression in 8 pairs of clinical samples through immunoblotting technique and found that MIB2 expression in lung cancer tissue was also up regulated compared with the adjacent normal tissue (Fig. 2a and b). Besides, we detected the MIB2 expression in 65 pairs of lung cancer using Real-time PCR and found that MIB2 was significantly up regulated in lung cancer tissue compared with the adjacent normal lung tissue (P = 0.0007) (Fig. 2c; Table 1). In addition, higher expression of MIB2 was obviously related to distal metastasis (P = 0.0393) and clinical stage (P = 0.0174) (Fig. 2d and e).

**Table 1:** Relationship between MIB2 expression and clinicopathologic characteristics in 65 NSCLC patients

Characteristics	Number of cases(%)	MIB2 expression	
		Mean ± SD	P value
Age			
≤ 60	26 (40)	0.0067 ± 0.0051	0.111
$>$60	39 (60)	0.0053 ± 0.0018	
Gender			
Female	26 (40)	0.0062 ± 0.005	0.5121
Male	39 (60)	0.0056 ± 0.0021	
Tumor size(cm)			
≤ 3	17 (26)	0.0047 ± 0.0023	0.1655
$>$3	48 (74)	0.0062 ± 0.0039	
Tissue			***
NSCLC	65	0.0033 ± 0.0036	0.0007
Noncancerous	65	0.0057 ± 0.004	
Clinical stage			*
I + II	31 (48)	0.0046 ± 0.0021	0.0174
III + IV	34 (52)	0.0068 ± 0.0045	
Metastasis			*
No	30 (46)	0.0047 ± 0.0021	0.0393
Yes	35 (54)	0.0067 ± 0.0045	

*P < 0.05, **P < 0.01 and ***P < 0.001

The bold formatting used in the table was considered to have a significant difference

### MIB2 is essential for NSCLC cell proliferation

**Fig. 3:**
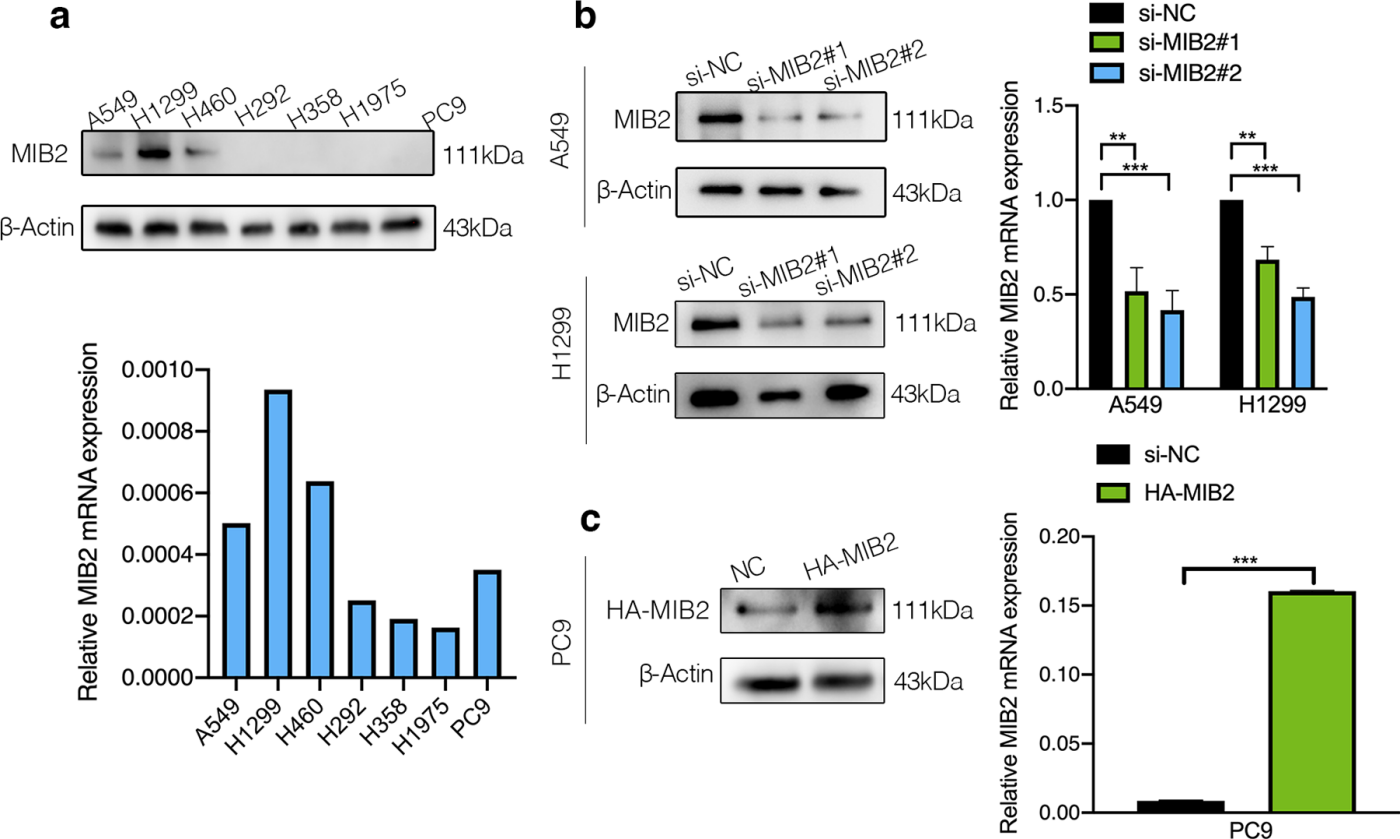
Efficiency of MIB2 knockdown and overexpression tested by qRT-PCR and immunoblotting analysis. (**a**) Western blot and Real-time PCR analysis were used to quantify the levels of MIB2 in seven NSCLC cell lines. (**b**) The knockdown efficiency of cells after transfection with si-MIB2 in A549 and H1229 cell lines, respectively. (**c**) The overexpression efficiency of cells after transfection with lentivirus in PC9 cells. *p < 0.05; **p < 0.01; ***p < 0.001

**Fig. 4:**
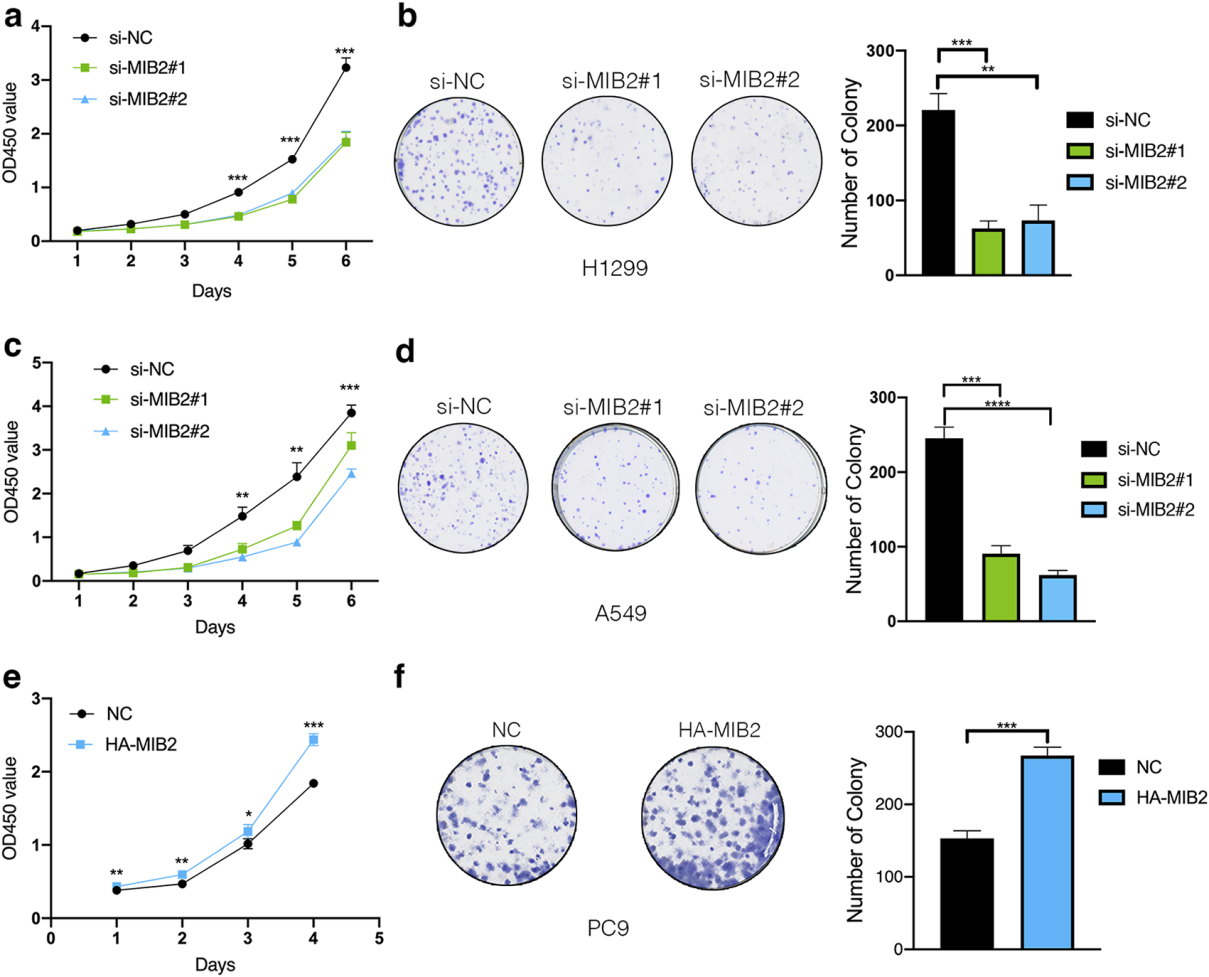
Effect of MIB2 on proliferation of NSCLC. (**a-b**) Effects of MIB2 on proliferative abilities of A549 and H1299 cell lines were detected by CCK-8 assay. (**c**) Effects of MIB2 on proliferative abilities of PC9 cells were detected by Colony formation assay

**Fig. 5:**
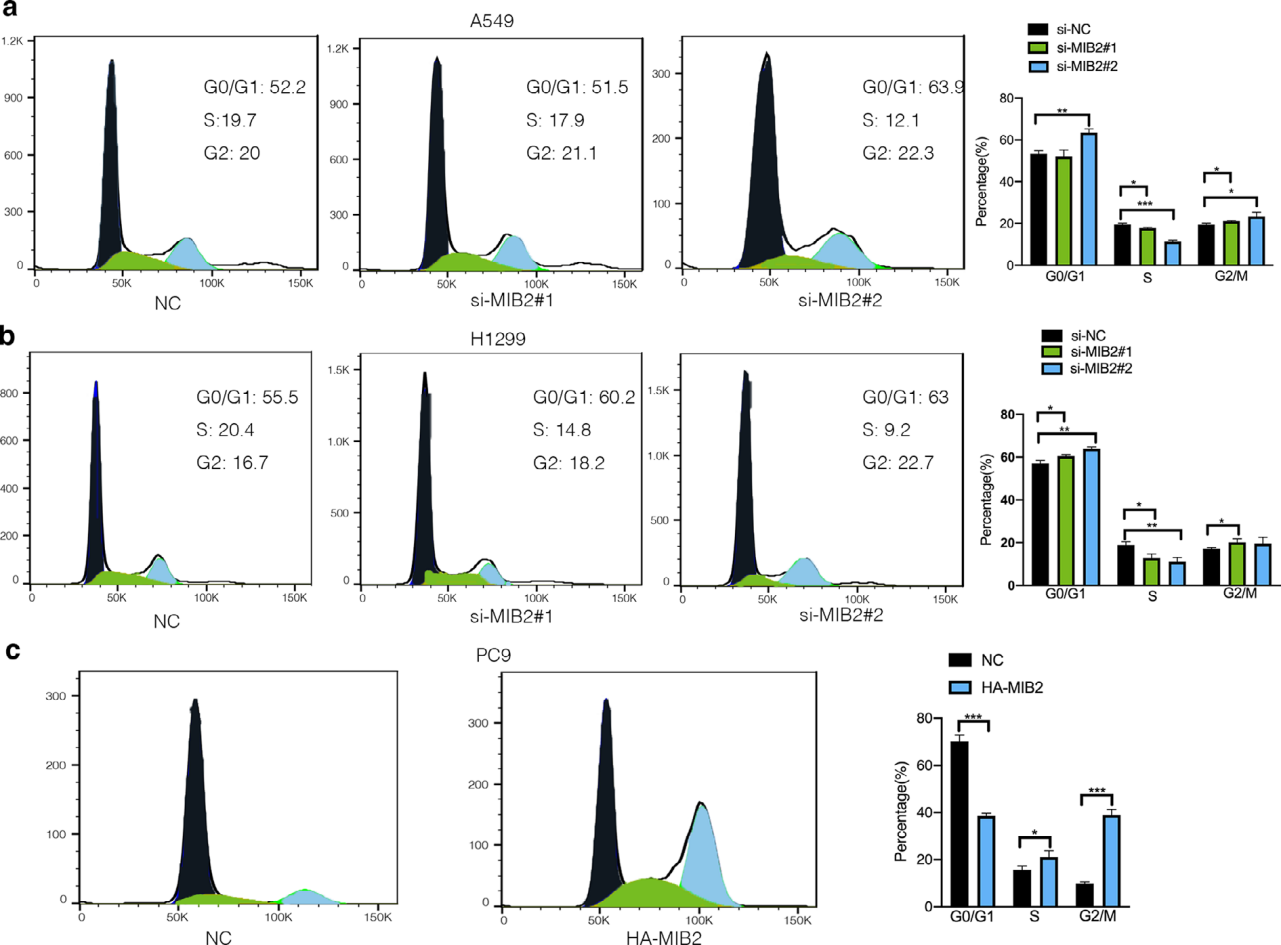
Effects of MIB2 on cell cycle. (**a-b**) Flow cytometric analysis shows the effects of MIB2 knockdown on cell cycle of A549 and H1229 cells. (**c**) Flow cytometric analysis shows the effects of MIB2 overexpression on cell cycle of PC9 cells. *p < 0.05; **p < 0.01; ***p < 0.001

The RNA and protein levels of MIB2 were examined in NSCLC cell lines including A549, H1299, H460, H292, H358, H1975, and PC9. As shown in Fig. 3a, MIB2 expression was relatively higher in A549, H1299, and H460. What’s more, expression of MIB2 in PC9 cells was relatively low. To investigate the role of MIB2 in NSCLC, we knocked down endogenous MIB2 expression by transient transfecting two si-RNAs in both of A549 and H1299 cells. Besides, we also overexpressed MIB2 in PC9 cells. The efficiency of knockdown and overexpression is tested by qRT-PCR and immunoblotting analysis (Fig. 3b and c). CCK8 and colony formation assays showed that downregulation of MIB2 significantly inhibits the cell proliferation (Fig. 4a and c) and colony formation abilities (Fig. 4b and d). Overexpression of MIB2 enhanced the ability of proliferation of PC9 cells (Fig. 4e and f). To detect that whether the observed effects on cell proliferation are specific to MIB2 or whether they are due to off-target effects of the siRNAs used to knock down MIB2 expression. We established two groups of cells in A549 and H1299 cell lines, which were stable knockdown MIB2 and stable knockdown MIB2 followed by overexpression of MIB2(Figure S1a&d). After conducting CCK8 assay, we found that MIB2 knockdown inhibited lung cancer cell proliferation, while overexpression of MIB2 can save the decrease of cell proliferation caused by MIB2 knockdown (Figure S1b,S1e). Clone assays also showed that MIB2 knockdown inhibited lung cancer clone ability, while MIB2 overexpression could rescue the inhibition of lung cancer proliferation (Figure S1c,S1f). Moreover, we detected cell cycle by flow cytometry. MIB2 silence was followed by an arrest in G0/G1 phase in H1299 cell line in A549 cell line (Fig. 5a and b). Overexpression of MIB2 was followed by a significant increase in the number of cells in S and G2/M phase (Fig. 5c).

### MIB2 is required for NSCLC migration and invasion

**Fig. 6:**
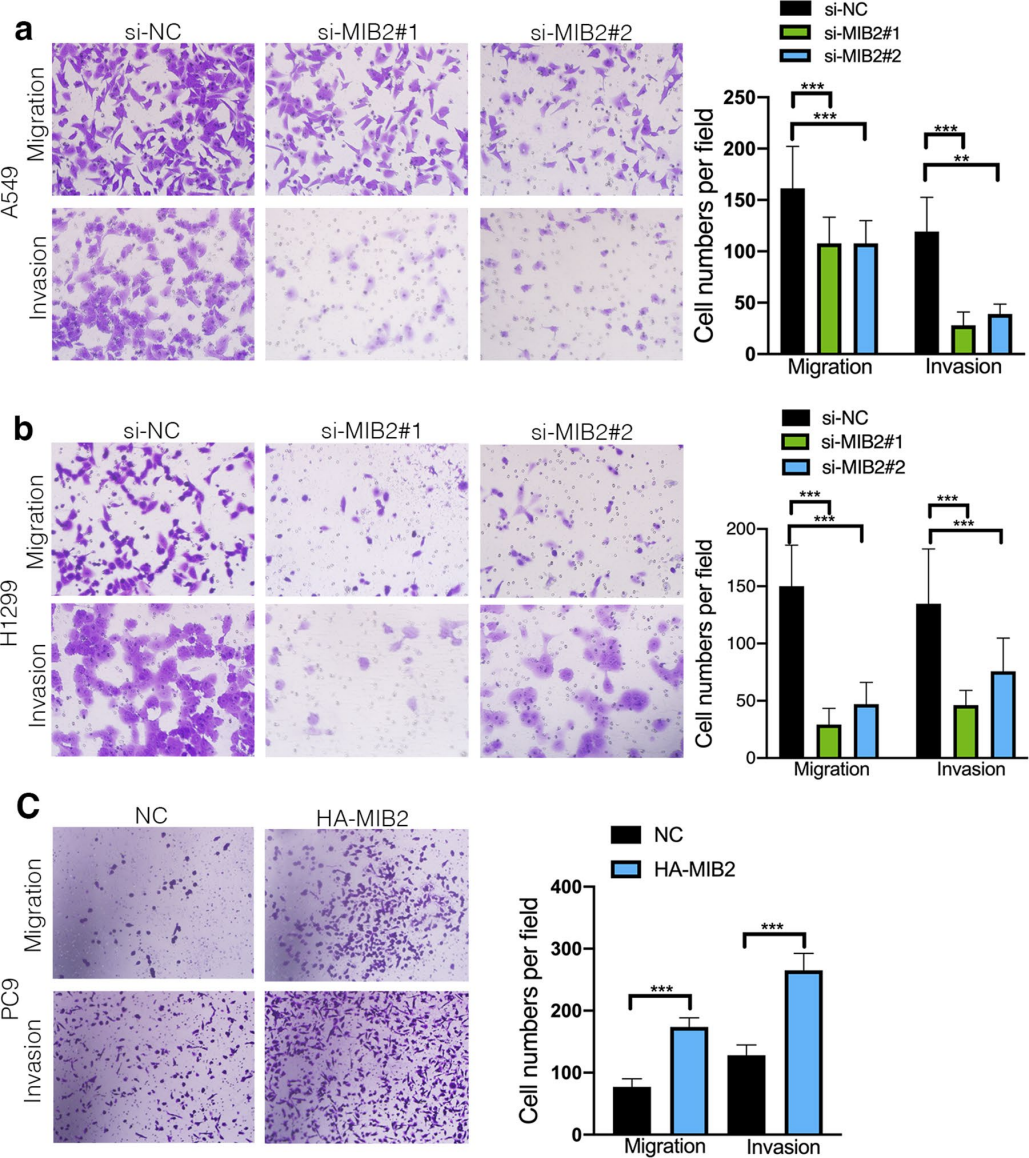
Effects of MIB2 on metastasis and invasion of NSCLC. (**a-b**) Transwell assays in A549 and H1299 cell transfected with two independent MIB2 siRNAs. (**c**) Transwell assays in PC9 cells with overexpression of MIB2. *p < 0.05; **p < 0.01; ***p < 0.001

To determine potential MIB2-promoting functions in NSCLC, we used transwell assays to investigate the role of MIB2 in NSCLC migration and invasion (Fig. 6a and b). We observed that cell migration and invasion abilities were substantially impressed after MIB2 knockdown. Besides, enhanced abilities of cell migration and invasion can be seen in PC9 cells after MIB2 overexpression (Fig. 6c). What’s more, the wound-healing assays showed that knockdown of MIB2 decreased migration rates of A549 and H1299 cell compared with controls in A549 and H1299 cells (Figure S2). Together, these results demonstrate that MIB2 plays an important role in promoting the migration and invasion of NSCLC cell lines in vitro.

### MIB2 facilitates tumorigenesis by regulating cell cycle

**Fig. 7:**
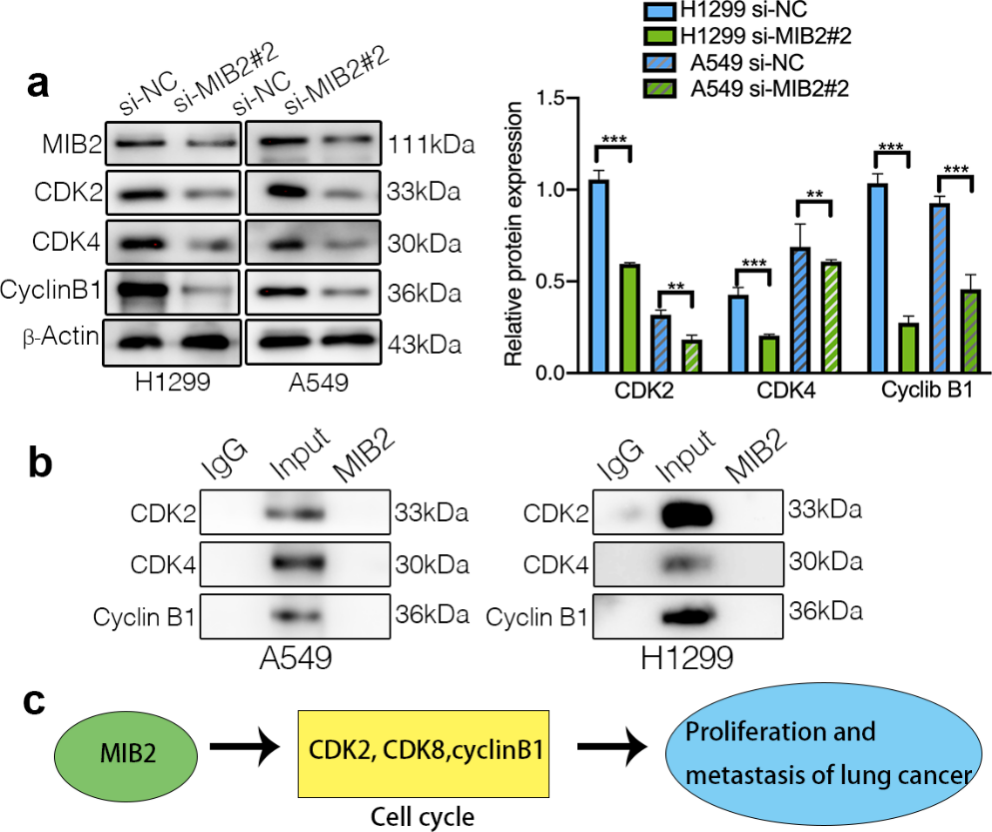
Validation of MIB2 effects on cell cycle by western blot. (**a**) Western blot analysis to quantify the effect of MIB2 knockdown in cell cycle proteins in A549 and h1299 cell lines. (**b**) The binding relationship between MIB2 and CDK2, CDK4, Cyclin B1 verified by IP assay. (**c**) Diagram of possible mechanism of MIB2. *p < 0.05; **p < 0.01; ***p < 0.001

To explore the molecular mechanisms of MIB2-induced cell cycle arrest, we detected expression of cell cycle proteins in MIB2 knockdown cells by immunoblotting detection technology (Fig. 7a). We observed that the expression level of CDK2, CDK4, and Cyclin B1 were reduced correlated with the knockdown of MIB2. We conducted IP assay and found that MIB2 linked to CDK2, CDK4, and Cyclin B1 indirectly (Fig. 7b). We suggest that MIB2 indirectly regulate the proliferation of lung cancer cells by regulating these cell cycle-related proteins. Figure 7c shows the potential mechanism of MIB2 regulating lung cancer proliferation.

## Discussion

Mind boom family proteins comprise of MIB2 and MIB1, both of which belong to the E3 ligase family and participate in regulating Notch signaling activation(Koo et al. 2007). Studies have found that E3 ligase is related to the regulation of lung cancer cell proliferation, metastasis, and treatment resistance recent years. As a member of the E3 ligase family, MIB2 has gradually received attention in research these years. Previously, MIB2 has been reported down or up regulation in different kinds of tumors. For example, MIB2 is reported up-regulated in leiomyoma, while MIB2 acts as a tumor suppressor in melanoma(Sahar et al. 2019; Takeuchi et al. 2003, 2005a, b). It is worth noting that MIB2 is reported to be a suppressor in lung cancer, which discover that MIB2 inhibits the proliferation, migration, and invasion of non-small cell lung cancer through ubiquitination of Notch1(Guo et al. 2022). We explored the expression of MIB2 in lung cancer samples and normal lung tissue in public databases including GEO and TCGA database, which was a strong evidence that MIB2 promoted the development of lung cancer. Based on these results, we detected MIB2 expression in NSCLC cell lines and clinical lung cancer tissue collected by ourselves. We assumed that MIB2 may exert in NSCLC progression as well. Then, we found that MIB2 was up regulated in NSCLC samples compared with normal tissue. We confirmed the promoting role of MIB2 in the occurrence and proliferation of lung cancer through proliferation and cloning experiments. Then, we performed the transwell and wound healing assay to study the role of MIB2 on the metastasis and invasion of lung cancer. Knockdown of MIB2 significantly attenuates the metastatic and invasive ability of A549 and H1299 lung cancer cell lines.

MIB2 is reported in participating the process of many signaling pathway regulation. For example, MIB2 involves in the NF-κB signaling, T-cells signaling, NOTCH signaling activation, and interferon signaling(Ben Khalaf et al. 2019; Koo et al. 2005; Mikami et al. 2015; Piccolo et al. 2017; Stempin et al. 2011; Uematsu et al. 2019; Ye et al. 2014). We noticed that MIB2 was reported to play a critical role in deciding the cell apoptosis through ubiquitylation of cFLIP(L) or by suppressing cytotoxic potential of RIPK(Feltham et al. 2018; Nakabayashi et al. 2021). In addition, it is intriguing that MIB2 is also reported to regulate cell apoptosis through B cells in glioma(Bai et al. 2018). Based on these studies, we investigated the effect of knockdown of MIB2 on cell cycle regulation by flow cytometry analysis. Knockdown of MIB2 can significantly promote the apoptosis of lung cancer cells, which indicates that MIB2 may play a role in the occurrence and development of lung cancer by inhibiting apoptosis. To further verified the role of MIB2 in modulating cell cycle, we examined the key proteins associated with cell cycle modulation in A549 and H1299. The results demonstrated that MIB2 knockdown led to low expression of cyclins and CDKs including CDK2, CDK4, and cyclinB1. According to previous studies, accumulation of CDK2 as well as cyclinA are activated to make sure the initiation of replication and the entrance to S phase. Accumulation of cyclinA/B-CDK1 complex allows the mitotic entry. CyclinD-CDK4/6 participates in preventing cell cycle exit and allowing the next cell cycle(Matthews et al. 2022). The accordant variations between MIB2 and cyclins confirm the promoting role of MIB2 in lung cancer cells and remind us that MIB2 may activate the progression of lung cancer through promoting the accumulation of cell cycle complexes.

However, our studies only include limited clinical samples, so we will try to expand the size of the samples and make the results more validate in the future. What’s more, our results are still not clear about the mechanism by which MIB2 regulates cell cycle, which needs more studies to focus on this field.

## Conclusion

Collectively, MIB2 is significantly up regulated in lung cancer tissue and is positively correlated with poor prognosis of non-small cell lung cancer patients. MIB2 promotes the proliferation, migration, and invasion of lung cancer cell lines by regulating cyclins, CDKs and EMT-related proteins. The sophisticated regulatory mechanism needs more in-depth study. MIB2 may be an important clinical marker and may serve for precise targeted therapy in non-small cell lung cancer.

## Electronic Supplementary Material

Below is the link to the electronic supplementary material.

Supplementary Material 1

Supplementary Material 2
