# Supplementary figures and images for: MIB2 promotes the progression of non-small cell lung cancer by regulating cell cycle control pathways
Source: Genes Genomics. 2023 Jul 12;45(9):1143–52. doi: 10.1007/s13258-023-01423-4 (PMC10435422; doi:10.1007/s13258-023-01423-4)

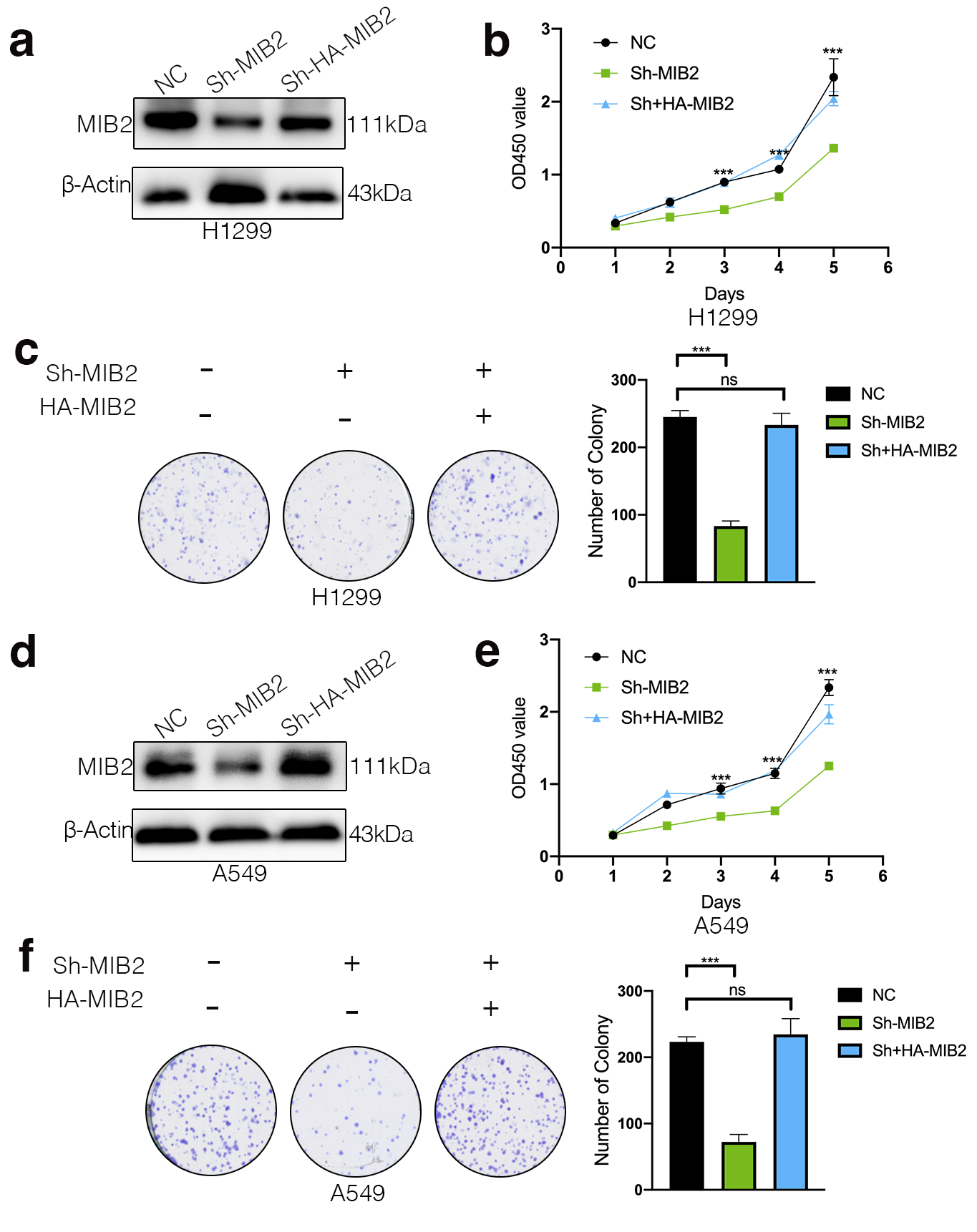

Supplement: Supplementary file 1 — Supplementary Material 1 [file 13258_2023_1423_MOESM1_ESM.png]

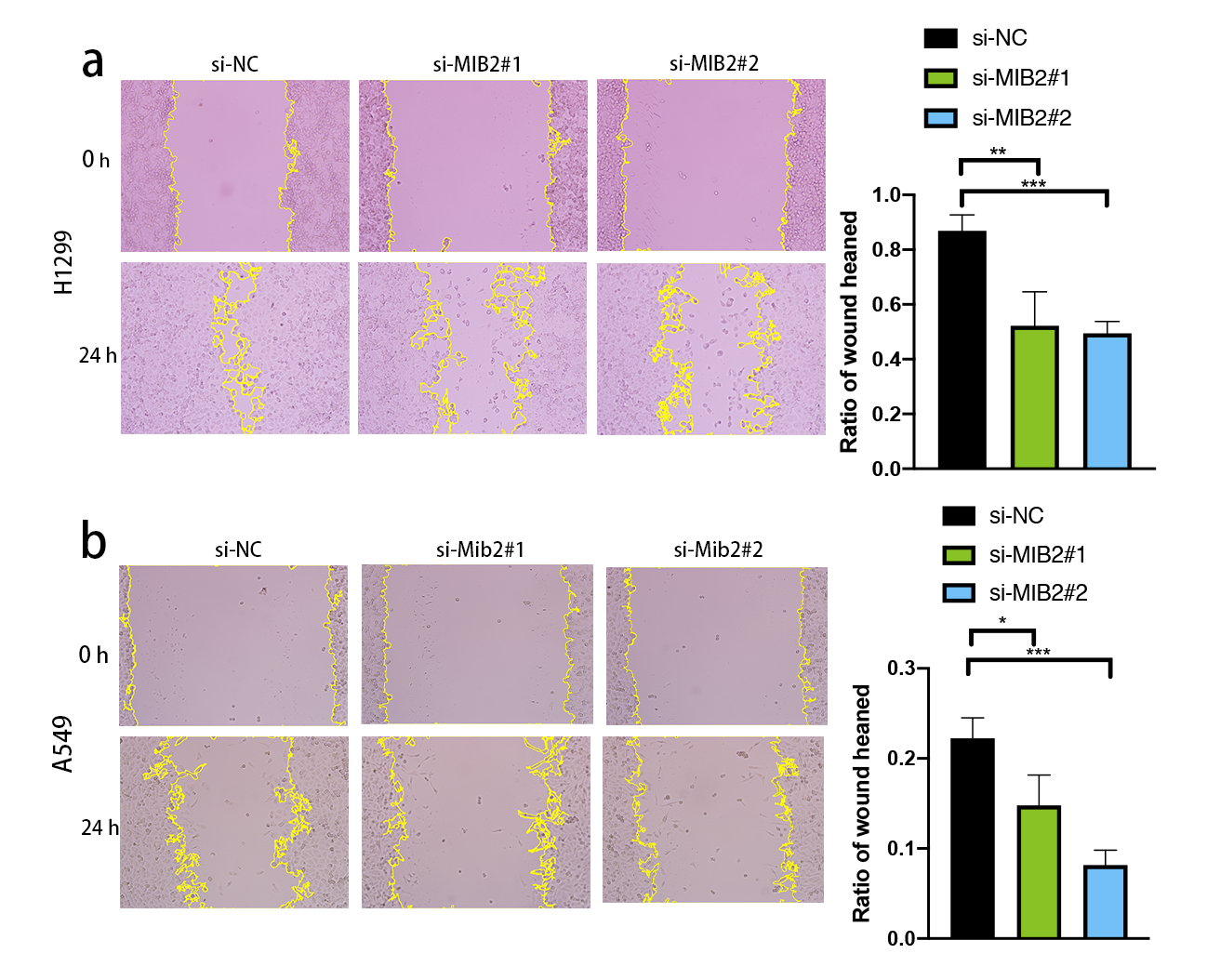

Supplement: Supplementary file 2 — Supplementary Material 2 [file 13258_2023_1423_MOESM2_ESM.png]
